# Retained Foreign Body in a Diabetic Patient’s Hand

**DOI:** 10.2174/1874325001812010203

**Published:** 2018-06-14

**Authors:** Frank Liaw, Odhrán Murray, Yan Yu Tan, Timothy Hems

**Affiliations:** 1 Queen Elizabeth University Hospital, Trauma & Orthopaedics, Glasgow, Scotland; 2 Royal Stoke University Hospital, General Surgery, Newcastle-under-Lyme, Newcastle, England

**Keywords:** Diabetic neuropathy, Foreign body retention, Osteomyelitis, Diabetes mellitus, Case report

## Abstract

**Background:**

Diabetic peripheral neuropathy puts patients at increased risk of acute injury by foreign bodies and also contributes to delayed presentation and diagnosis.

**Case report:**

We describe a 57-year-old patient with poorly controlled type 1 diabetes who presented with a three-week history of worsening swelling and erythema in the metacarpophalangeal joint of his left thumb. He denied any previous trauma or injury and was initially treated with intravenous antibiotics. Subsequent imaging revealed septic arthritis and osteomyelitis secondary to a retained foreign body, which was surgically removed in theatre.

**Conclusion:**

This is the first reported case of a retained foreign body in the hand of a diabetic patient, and demonstrates the importance of early radiological imaging of peripheral limb injuries in high-risk patients.

## 1 INTRODUCTION 

Diabetic peripheral neuropathy results in a disruption of normal protective sensory mechanisms. Of particular interest to orthopaedics, the development of diabetic peripheral neuropathy can be a precursor of neuropathic arthropathy, also known as Charcot joints, and chronic ulceration, a resulting infection of which would potentially require amputation [1]. This loss of sensation also puts patients at risk of acute injury by foreign bodies. Further exacerbated by a late presentation, compromised immune system and poor wound healing, penetration and retention of a foreign body may not be recognised until it reaches a stage where surgical intervention is required.

## 2 CASE REPORT

We present the case of a 57-year-old patient with a three-week history of worsening swelling and erythema in the metacarpophalangeal joint of his left thumb. The patient denied having sustained any trauma or injury to his hand prior to the onset of symptoms.

He had initially presented to his general practitioner, who commenced him on a course of oral flucloxacillin for suspected cellulitis. Of note in his past medical history is poorly controlled type 1 diabetes mellitus with bilateral diabetic retinopathy and neovascular glaucoma.

On examination, he was apyrexial and systemically stable. Closer inspection of his left thumb demonstrated significant swelling to the metacarpophalangeal joint and a discharging wound over the interphalangeal joint. The interphalangeal joint was fixed in flexion, with tenderness noted over the dorsum of the joint. There was altered sensation in both hands and feet, in a glove and stocking distribution consistent with diabetic neuropathy, as well as a chronic heel ulcer present on his right foot. Admission blood investigations testified to his poor diabetic control, with random serum glucose of 35 mmol/L and HbA1c of 119 mmol/mol. Inflammatory markers were mildly elevated with a C-reactive protein level of 14 mg/L and leucocytes of 12 x10^9^/L.

**Fig. (1):**
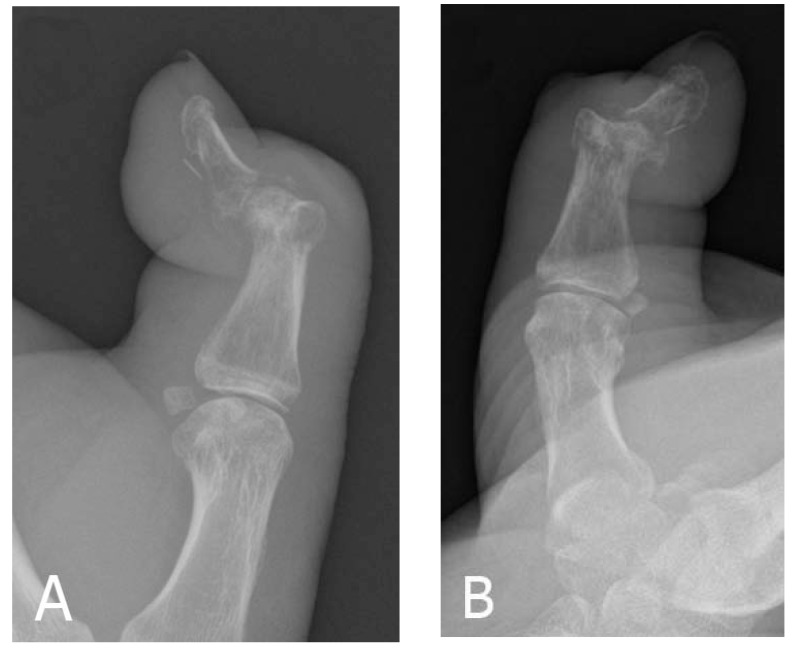
(a) Lateral and (b) anteroposterior radiographs of the left thumb showing a 4mm linear density anterior to the distal phalanx in addition to erosive/destructive changes in keeping with septic arthritis and adjacent osteomyelitis along with a pathological fracture of the neck of the proximal phalanx.

He was admitted for treatment with intravenous fluids and a course of intravenous flucloxacillin and clindamycin for cellulitis. An insulin sliding scale was commenced due to his erratic blood glucose. Eight days after admission, there was slight improvement noted in the swelling and erythema of his thumb; however, the inflammation did not completely resolve. A plain radiograph of his left thumb was obtained and this demonstrated erosive/destructive changes in keeping with septic arthritis and adjacent osteomyelitis along with a pathological fracture of the neck of the proximal phalanx (Fig. **1**).

Moreover, a foreign body was identified on the volar aspect of the distal phalanx, which the patient denied having any knowledge of.

**Fig. (2):**
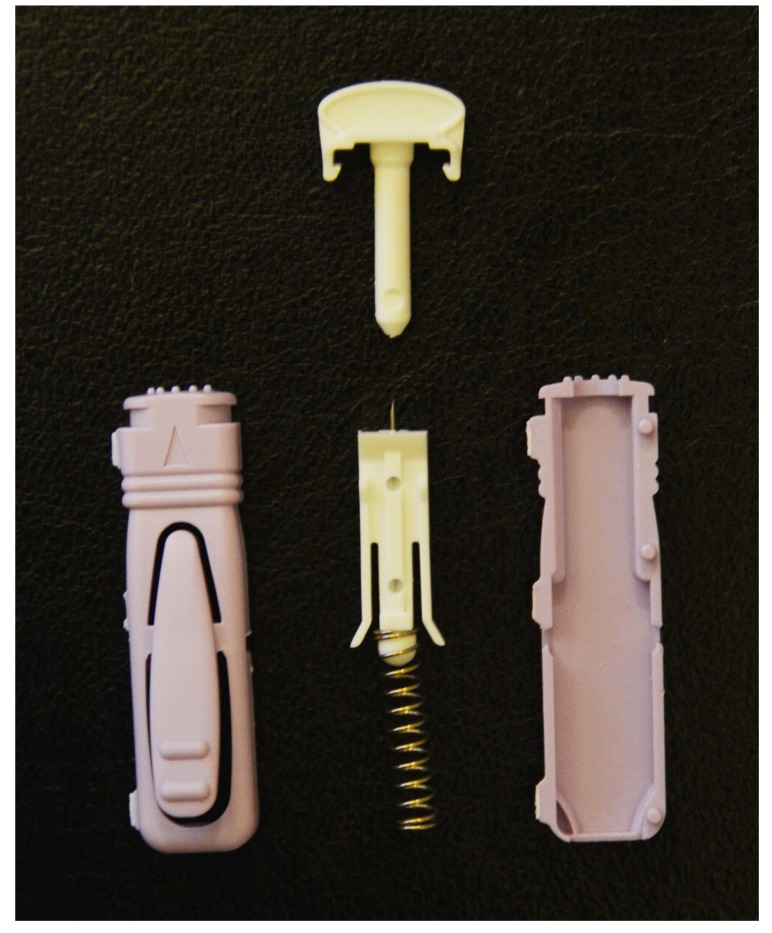
A dismantled single use safety lancet ordinarily used for blood glucose measuring. Featured here is a Unistik 3 Comfort – the same model the patient utilises. Unfortunately the actual needle tip retrieved from the patient’s left thumb was not photographed prior to disposal at time of surgery.

In view of the failure of the infection to resolve with conservative management, especially in the presence of a foreign body, the patient was listed for surgical debridement. Intraoperative findings included necrotic bone on either side of the joint with no frank pus and a needle fragment in the palmar surface of the distal phalanx. The needle fragment was removed, and debridement and washout of the necrotic bone and adjacent soft tissue were performed. The extracted needle tip was similar to a broken lancet needle Unistik 3 Comfort the patient usually uses for obtaining a blood glucose reading (Fig. **2**).

Postoperatively, he continued on intravenous antibiotics, with a plan to consider amputation if there was any further deterioration in his condition. Tissue samples of bone and soft tissue obtained intraoperatively grew *Klebsiella oxytoca*, *Staphylococcus aureus*, and *Candida albicans*. Following discussion with Microbiology, the patient was commenced on a two-week course of intravenous flucloxacillin, oral ciprofloxacin and fluconazole. The osteomyelitis was noted to settle during his admission, and no further surgery was deemed necessary. The patient was discharged on a three-month course of oral antibiotics, but did not attend his review appointments and has subsequently been lost to follow up.

## 3 DISCUSSION

Given the incidence of diabetes mellitus in the population, there is a surprising paucity of reported cases in the literature demonstrating foreign body retention in patients with diabetes. Nadig *et al.* noted just six cases of patients with diabetic sensory neuropathy and insulin needle fragments found in the feet, abdomen, and shoulder, with one of these patients requiring an amputation of the great toe due to osteomyelitis [2]. Woolfrey *et al.* illustrated a further case of insulin needle fragment retention in which there were no clinical signs of foreign body or infection [3]. To our knowledge, there have been no cases reported in the literature demonstrating retention of a foreign body in the hand of a patient with diabetic neuropathy.

Injuries involving foreign bodies in the hand are generally common, and retained foreign bodies carry several potential complications including infection, migration, pain, stiffness and granuloma formation [4]. In this diabetic patient with no history of trauma or injury, the initial diagnosis was of cellulitis. As such, it was not routine practice in our hospital to obtain plain radiographs of the affected area. The decision to obtain plain radiographs was only prompted by a failure to improve following a course of intravenous antibiotics, raising suspicion of an alternate diagnosis or underlying cause. The initial index of suspicion should, therefore, be high in any known or suspected hand injury, especially in high-risk patients with diabetic neuropathy, in whom cases of the retained foreign body may be more common than the literature suggests.

In these cases, the literature recommends obtaining plain radiographs with multiple views in the first instance. In addition, ultrasound imaging may also be useful, particularly in the detection of radiolucent foreign bodies and to guide removal of foreign objects not readily found at surgery. Computed tomography and magnetic resonance imaging are the best studies to evaluate complications that are associated with retained foreign bodies such as infection or damage to surrounding structures [5, 6].

To minimise the risk of lancet needle retention as an unintentional consequence of blood glucose monitoring, perhaps the most promising and definitive solution would be the development of non-invasive glucose monitoring. Currently numerous academic institutions and commercial companies are working intensely on various technologies such as bio-impedance spectroscopy, fluorescent technology and near-infrared spectroscopy; however, there is no non-invasive glucose system available yet that has documented usability and reliability under conditions of daily life [7]. In the meantime, therefore, educating all diabetic patients on self-examination of distal limbs would be the primary preventive measure.

## CONCLUSION

In conclusion, our case demonstrates the potential risk of lancet needle retention in diabetic patients’ hands and highlights the value of early radiological imaging of localised peripheral limb swelling in high-risk patients with diabetes.
